# Red Wine Polyphenols for Cancer Prevention

**DOI:** 10.3390/ijms9050842

**Published:** 2008-05-20

**Authors:** Shan He, Cuirong Sun, Yuanjiang Pan

**Affiliations:** Department of Chemistry, Zhejiang University, Hangzhou 310027, P. R. China

**Keywords:** Red wine polyphenols, cancer prevention, carcinogenesis, aromatase inhibitor, myricetin

## Abstract

Conventional cancer therapies, the second leading cause of death worldwide, result in serious side effects and, at best, merely extend the patient's lifespan by a few years. Searching for effective prevention is of high priority in both basic and clinical sciences. In recent decades natural products have been considered to be an important source of cancer chemopreventive agents. Red wine polyphenols, which consisted of various powerful antioxidants such as flavonoids and stilbenes, have been implicated in cancer prevention and that promote human health without recognizable side effects. Since resveratrol, a major component of red wine polyphenols, has been studied and reviewed extensively for its chemopreventive activity to interfere with the multi-stage carcinogenesis, this review focuses on recent progress in studies on cancer chemopreventive activities of red wine polyphenol extracts and fractions as well as other red wine polyphenols, like procyanidin B5 analogues and myricetin.

## 1. Introduction

Cancer is one of the main reasons of death in both men and women, claiming over 6 million people each year worldwide. Chemoprevention, a relatively new and promising strategy to prevent cancer, is defined as the use of either natural or synthetic substances or their combinations to block, reverse or retard the process of carcinogenesis [1]. In spite of substantial progress in the development of anticancer therapies, the incidence of cancer is still increasing worldwide. Carcinogenesis is generally recognized as a multi-step process in which distinct molecular and cellular alterations occur. From the studies of experimentally induced carcinogenesis in rodents, its development is considered to consist of several separate, but closely linked, stages — initiation, promotion and progression. And it takes many years to turn into complete malignancy. Therefore, there are ample opportunities to intervene in the development of cancer before the onset of malignancy.

A diet rich in vegetable and fruit reduces the risk of cancer [2]. Polyphenols in these foods and beverages are thought to be among the constituents responsible for the reduced cancer risk because they are protective in cell cultures and in animals pretreated with carcinogenic chemicals [3–9] or cancer cells [10]. Grapes and red wine are an abundant source of polyphenols and represent an important dietary component for some populations. In recent years, a large body of literature has been devoted to studies describing the potential cancer chemopreventive activities of red wine polyphenols [11, 12], which have been shown to block carcinogenesis and to inhibit the growth of tumors in whole animals or in cell culture. In many instances, these effects can be attributed to plausible biochemical mechanisms including enhanced apoptosis, growth arrest at one or more phases in cell cycle, inhibition of DNA synthesis, and modulation of signal transduction pathways by altered expression of key enzymes such as cyclooxygenases and protein kinases [13, 14]. Moreover, consumption of polyphenols from wine could account for the lower risk of rectal cancer among wine drinkers, compared to consumers of beer and spirits [15].

Since a pioneering study by Pezzuto and colleagues [16], resveratrol, a major component of red wine polyphenols, has been studied and reviewed extensively for the chemopreventive activity to interfere with the multistage carcinogenesis [17, 18]. However, resveratrol was not believed to be the only phytochemical that contribute to the chemopreventive activity of red wine. In this review we focus on recent progress in studies on cancer chemopreventive activities of red wine polyphenol extracts and fractions as well as other red wine polyphenols.

## 2. Cancer chemopreventive activities of red wine polyphenol extracts and fractions

In recent years, experimental studies have shown that polyphenols from red wine, like resveratrol [16], quercetin [19], (+)-catechin [20] and gallic acid [21], were potential cancer chemopreventive agents. However, red wine contains a wide range of different polyphenols [22] and protective effects have not been assigned to a specific fraction or compound, so it is not yet clear which compounds present in red wine are endowed with protective activity. Therefore, to investigate the cancer chemopreventive effects of red wine polyphenols as total extract or fractions are of importance.

In 1996, Clifford and coworkers investigated the cancer chemopreventive activities of dehydrated-dealcoholized red wine solids, which were consumed as part of a precisely defined complete diet. Sibling transgenic mice were weaned onto an amino acid-based diet alone or supplemented with red wine solids. The supplemented diet was fed continuously for three generations to ensure that it supported normal growth and reproduction. Red wine solid supplement was reported to delay tumor onset, that intact catechin was absorbed, and that the supplemented diet supported normal growth and reproduction for three generations. Therefore, red wine solids may be a useful dietary supplement for animal studies and human clinical trials [23].

In 2000, Caderni and coworkers made a comparison among extracts from black and green tea and red wine of their effects on azoxymethane (AOM) induced intestinal carcinogenesis. Male F344 rats were treated with AOM and then allocated into groups receiving black tea, green tea or red wine extracts mixed in their diet. In the rats treated with black tea or wine extracts, there were significantly fewer colorectal tumours than in controls. Significantly fewer rats in the black tea and wine extract groups had adenomas than in controls. The tumours from the black tea group and, to a lesser extent, those from the wine group, had a significantly greater apoptotic index than tumours in controls. In contrast, the apoptotic index of the normal mucosa did not vary among groups. These findings indicated that black tea and wine extracts, but not green tea extracts, could protect against AOM-induced colon carcinogenesis by a mechanism probably involving increased apoptosis in tumours [24]. In continuation, the research group recently investigated the effect of high molecular weight polyphenols (HMWP), low molecular weight polyphenols (LMWP) and total polyphenolic extracts from red wine (WE) on colon carcinogenesis. Their results demonstrated that total WE, but neither the HMWP nor the LMWP, have inhibitory effect on the process of colon carcinogenesis induced by dimethylhydrazine (DMH). These conclusions were in accordance with previous observations, showing that HMWP do not inhibit aberrant crypt foci (ACF), putative preneoplastic lesions [25]. They suggested that a protective effect of wine polyphenols is better observed administering a complex mixture [26].

In 2002, Briviba and coworkers showed that red wine polyphenols inhibited the proliferation of transformed colon epithelial cells HT 29 clone 19A induced by epidermal growth factor (EGF). Inhibition of proliferation was also associated with modulation of activation of mitogen-activated protein kinases (MAPK). Stress activated c-Jun N-terminal kinases 1/2 (JNK) and p38 MAPK were significantly activated by red wine polyphenols. Maximum phosphorylation of MAPK was observed after treatment with red wine polyphenols. Furthermore, activation of extracellular signal regulated kinase (ERK) 1/2 by EGF was significantly inhibited by red wine polyphenols. This signaling pattern, activation of JNK 1/2 and p38 MAPK and inhibition of ERK 1/2, is typical for anti-proliferative compounds, indicating that red wine polyphenols may inhibit the proliferation of colon carcinoma cells by modulating MAPK intracellular signal transduction pathways [27].

**Figure 1.**
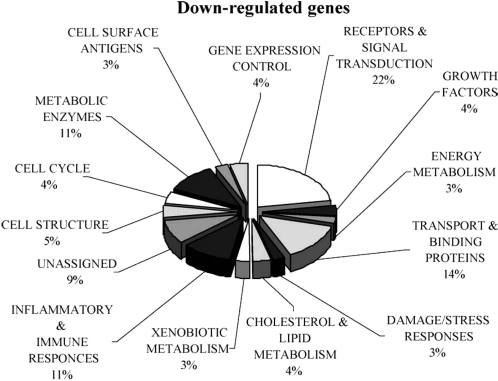
Diagram of the down-regulated genes after polyphenol-treatment and their function. The % indicated the % frequency of genes in a functional class relative to the total amount of genes varied by the treatment [28].

In 2005, Dolara and coworkers reported that red wine polyphenols administered with the diet to F344 rats for 16 weeks inhibited colon carcinogenesis induced by AOM or DMH. Furthermore the molecular effects of wine polyphenols were investigated by the microarray technology to study gene expression profiles. Global expression analysis of 5707 genes revealed an extensive down-regulation of genes involved in a wide range of physiological functions, such as metabolism, transport, signal transduction and intercellular signaling (Figure 1). It was observed that two major regulatory pathways were down-regulated in the colon mucosa of polyphenols-treated rats: inflammatory response and steroid metabolism. Furthermore, a down-regulation of many genes regulating cell surface antigens, metabolic enzymes and cellular response to oxidative stress was also observed. In conclusion, reduction of oxidative damage, modulation of colonic flora and variation in gene expression may all concur in the modulation of intestinal function and carcinogenesis by red wine polyphenols [28].

In a more recent study [29], the mechanism of selective cytotoxicity induced by red wine polyphenols against MCF-7 breast cancer cells was investigated in relation to their interference with calcium homeostasis. MCF-7 cells showed an increase in cytosolic calcium levels within 10 min of treatment with the polyphenols. MCF-7 cells treated with the red wine polyphenol fraction (RWPF) showed swelling of endoplasmic reticulum, dissolution of the nucleus, and loss of plasma membrane integrity as well as reduced mitochondrial membrane potential. These cells were arrested at the G2/M interphase. By contrast, MCF-10A cells did not show such changes after RWPF treatment. The results suggest that polyphenol-induced calcium release may disrupt mitochondrial function and cause membrane damage, resulting in selective cytotoxicity toward MCF-7 cells. This property could further be developed toward breast cancer prevention strategies either independently or in conjunction with conventional prevention therapies where a positive drug-nutrient interaction could be demonstrated.

## 3. Red wine polyphenols as aromatase inhibitors: potential preventive strategy of breast cancer

Estrogens play an important role in breast cancer development. Approximately 60% of premenopausal and 75% of postmenopausal patients have estrogen-dependent carcinomas. Aromatase, a cytochrome P450, is the enzyme that synthesizes estrogens by converting C19 androgens into aromatic C18 estrogenic steroids. In breast tumors, expression of aromatase is upregulated compared to that of surrounding noncancerous tissue [30–33]. Furthermore, estrogen produced *in situ* has a stronger influence than endogenous circulatory estrogen in breast tumor growth [34, 35]. Therefore, suppression of *in situ* estrogen formation in the breast of postmenopausal women by aromatase inhibitors is considered to be a useful approach for prevention and treatment of breast cancer.

**Figure 2.**
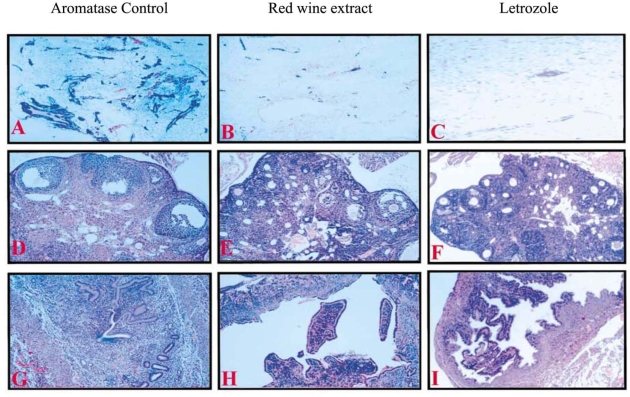
Red wine extract completely abrogated aromatase-induced hyperplasia and other changes in the mammary gland (B), ovary (E) and uterus (H), as compared to the control group (A, D and G), which showed comparable effects with Letrzole (C, F and I), a well known aromatase inhibitor. [37]

Enlightened by the finding that grape juice contained chemicals that act as potent inhibitors of aromatase [36], Eng and coworkers investigated aromatase inhibitory activities of grape wines [37]. Red wine was shown to be much more effective than white wine in suppression of aromatase activity (Table 1). C18 Sep-Pak cartridge (Waters Co.) separation of red wine extracts under an increasing acetonitrile (ACN) gradient found that the most active components were in the 20% ACN fraction, in that they inhibited the wild-type human placenta aromatase, wild-type porcine placenta and blastocyst aromatase in a dose-dependent fashion. The 20% ACN active fraction was heat stable and inhibited aromatase in a non-competitive manner. The aromatase-inhibitory action of red wine extracts was also examined with a transgenic mouse model in which aromatase is over-expressed in the mammary tissues. It was found that the intake of the 20% ACN fraction by gavage completely abrogated aromatase-induced hyperplasia and other changes in the mammary tissue (Figure 2). These results suggested that red wine or red wine extract might be a chemopreventive diet supplement for postmenopausal women who have a high risk of breast cancer [38].

**Figure 3.**
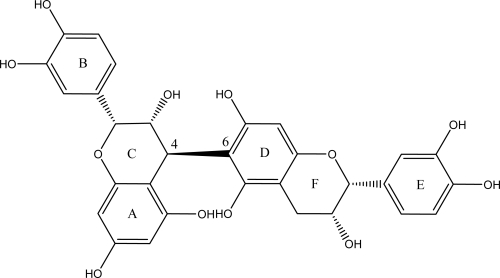
The chemical structure of procyanidin B5. Procyanidins B5–B8 are dimers with the 4–6 linkage and stereoisomers at position C-3.

In 2003, Eng and coworkers isolated the active compounds from the red wine fraction, which were identified as procyanidin B dimers (Figure 3) that were shown to be aromatase inhibitors [39]. Inhibition kinetic analysis on the most potent procyanidin B dimer has revealed that it competed with the binding of the androgen substrate with a *K_i_* value of 6 μM. Because mutations at Asp-309, Ser-378, and His-480 of aromatase significantly affected the binding of the procyanidin B dimer, these active site residues were thought to be important residues that interact with this phytochemical. The *in vivo* efficacy of procyanidin B dimers was evaluated in an aromatase-transfected MCF-7 breast cancer xenograft model. The procyanidin B dimers were able to reduce androgen-dependent tumor growth, indicating that these chemicals suppress *in situ* estrogen formation. These *in vitro* and *in vivo* studies demonstrated that procyanidin B dimers in red wine and grape seeds could be used as chemopreventive agents against breast cancer by suppressing *in situ* estrogen biosynthesis [39].

## 4. Cancer chemopreventive activities of myricetin

**Figure 4.**
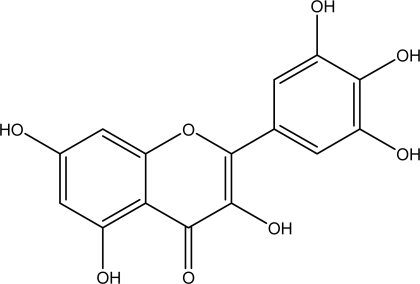
Chemical structure of myricetin.

Flavonols occur widely in nature in plants, including tea, berries, and vegetables [40]. The flavonol concentration in red wine is about 30 times higher that of resveratrol, and the major flavonol components in red wine are 3,3’,4’,5,5’,7-hexahydroxyflavone (myricetin, Figure 4) and 3,3’,4’,5,7-pentahydroxyflavone (quercetin) [22], which typically represent 20–50% of the total flavonol content [22]. Several studies have shown that myricetin exhibited anticarcinogenic activities. It was indicated that myricetin had a potent antioxidant capacity [41] and suppressed cancer development induced by polycyclic aromatic hydrocarbons in SENCAR mice [42]. It has also been demonstrated that myricetin exerted protective effects against two-stage skin tumorigenesis [43] and inhibited the growth of A549 lung cancer cells [44]. In colorectal cancer cells, myricetin inhibited the activity of matrix metalloproteinase-2 [45].

Recently, Lee and coworkers reported that myricetin inhibited 12-*O*-tetradecanoylphorbol-13-acetate (phorbol ester)-induced COX-2 expression in JB6 P+ mouse epidermal (JB6 P+) cells by suppressing activation of nuclear factor kappa B (NF-κB). Myricetin inhibited phorbol ester-induced upregulation of COX-2 protein, while resveratrol at the same concentration did not exert significant effects. The phorbol ester-induced production of prostaglandin E2 was also attenuated by myricetin treatment. Myricetin inhibited both COX-2 and NF-κB transactivation in phorbol ester-treated JB6 P+ cells. Myricetin blocked the phorbol ester-stimulated DNA binding activity of NF-κB. In addition, red wine extract inhibited phorbol ester-induced COX-2 expression and NF-κB transactivation in JB6 P+ cells. These results revealed that myricetin contributes to the chemopreventive effects of red wine through inhibition of COX-2 expression by blocking the activation of NF-κB [46].

Lee and coworkers subsequently reported that myricetin was a novel inhibitor of mitogen-activated protein kinase kinase (MEK)1 activity and transformation of JB6 P+ mouse epidermal cells [47]. Myricetin (10 μM) inhibited 12-*O*-tetradecanoylphorbol-13-acetate (TPA) or epidermal growth factor (EGF)-induced cell transformation by 76 or 72%, respectively, compared with respective reductions of 26 or 19% by resveratrol (20 μM). A combination of myricetin and resveratrol exerted additive but not synergistic effects on either TPA- or EGF- induced transformation. Myricetin, but not resveratrol, attenuated tumor promoter-induced activation of c-fos or activator protein-1. Myricetin strongly inhibited MEK1 kinase activity and suppressed TPA- or EGF- induced phosphorylation of extracellular signal-regulated kinase (ERK) or p90 ribosomal S6 kinase, downstream targets of MEK. Moreover, myricetin inhibited H-Ras-induced cell transformation more effectively than either PD098059, a MEK inhibitor, or resveratrol. Myricetin directly bound with glutathione *S*-transferase-MEK1 but did not compete with ATP. These results indicated that myricetin had potent anticancer-promoting activity and mainly targets MEK signaling, which may contribute to the chemopreventive potential of red wines.

## 5. Conclusions

Chemoprevention in combination with anticancer treatment represents an important approach to reduce morbidity and mortality from cancer. Red wines contain a large array of polyphenolic constituents that have been shown to block carcinogenesis and to inhibit the growth of tumors in whole animals, or in cell culture by altering the activity of certain enzymes or the expression of specific genes.

Red wine polyphenol extracts and fractions were reported to delay tumor onset in transgenic mice, inhibit azoxymethane (AOM) induced intestinal carcinogenesis by modulation of gene expression, inhibit epidermal growth factor induced the proliferation of transformed colon epithelial cells by modulation of activation of mitogen-activated protein kinases and show selective cytotoxicity against MCF-7 breast cancer cells. Supported by above exciting evidence, further investigation on cancer chemopreventive activities of red wine polyphenol extracts and fractions are strongly recommended.

Red wine polyphenols were found to be potent aromatase inhibitors, indicating potential treatment of breast cancer, since aromatase plays an important role in the carcinogenesis of breast cancer. The previous hypothesis that red wine had stronger anti-cancer activities than white wine was confirmed by the difference in inhibition of aromatase activity. An extensive study revealed that the procyanidin B dimers were identified to be the active principles. Further *in vitro* and *in vivo* studies demonstrated that procyanidin B dimers in red wine and grape seeds were potential chemopreventive agents against breast cancer by suppressing *in situ* estrogen biosynthesis.

Recent findings have demonstrated potent anticancer-promoting activity for myricetin, which mainly targeted MEK signaling and inhibited COX-2 expression by blocking the activation of NF-κB. Although resveratrol was believed to be a promising cancer chemopreventive agent from red wine, the search for other novel cancer chemopreventive polyphenols, like myricetin, is also of significance.

## Figures and Tables

**Table 1.** Inhibitory effect on human placental aromatase activity by complete red or white wines [38].

Wine	Percent remaining aromatizer activity
Red wine	
Cabernet Sauvignon, Tanglewood, 1996	0.29
Cabernet Sauvignon, Glen Ellen Prp Reserve, 1997	7.7
Cabernet Sauvignon, San Andrés, 1998	0.36
Merlot, JW Morris, 1997	0.42
Merlot, Forest Ville, 1997	0.46
Merlot, Hacienda, 1997	3.29
Merlot, Hacienda, 1998	0.9
Zinfande, Black Mountain, 1996	0.39
Zinfandel, Sequoia Ridge, 1996	0.39
Pinot Noir, Cambiaso, 1996	0.34
Pinot Noir, Hacienda, 1996	2.16
White wine	
Chardonnay Woodbridge, 1998	99.1
Chardonnay, Santa Rita Reserve, 1999	80
Fumé Blanc, Domaine Napa, 1996	112.5
Sauvignon Blanc, Turning Leaf, 1998	106.5
